# Clinical Features and Therapeutic Effects of Anti-leucine-rich Glioma Inactivated 1 Encephalitis: A Systematic Review

**DOI:** 10.3389/fneur.2021.791014

**Published:** 2022-01-12

**Authors:** Yuou Teng, Ting Li, Zhizhong Yang, Mingwan Su, Jingnian Ni, Mingqing Wei, Jing Shi, Jinzhou Tian

**Affiliations:** ^1^Dongzhimen Hospital, Beijing University of Chinese Medicine, Beijing, China; ^2^Department of Neurology, Dongzhimen Hospital, Beijing University of Chinese Medicine, Beijing, China

**Keywords:** anti-leucine rich glioma inactivated 1 encephalitis, LGI1, clinical features, diagnosis, treatment

## Abstract

**Background:** Clinical presentations and treatment programs about anti-leucine-rich glioma inactivated 1 (LGI1) encephalitis still remain incompletely understood.

**Objective:** This study analyzed the clinical features and therapeutic effects of anti-LGI1 encephalitis.

**Methods:** PubMed, EMBASE, and the Cochrane Library were searched to identify published English and Chinese articles until April 2021. Data were extracted, analyzed, and recorded in accordance with the Preferred Reporting Items for Systematic reviews and Meta-Analyses (PRISMA) guidelines.

**Results:** A total of 80 publications detailing 485 subjects matched our inclusion criteria. Short-term memory loss (75.22%), faciobrachial dystonic seizures (FBDS) (52.53%), other seizures excluding FBDS (68.48%), psychiatric symptoms (57.67%), and sleep disturbances (34.30%) were the most frequently described symptoms in anti-LGI1 encephalitis. Hyponatremia (54.90%) was the most common hematologic examination change. The risk of incidence rate of malignant tumors was higher than in healthy people. The positive rate of anti-LGI1 in serum (99.79%) was higher than CSF (77.38%). Steroids (93.02%), IVIG (87.50%), and combined use (96.67%) all had a high remission rate in the initial visit. A total of 35 of 215 cases relapsed, of which 6/35 (17.14%) did not use first-line treatment, and 21 (60.00%) did not maintain long-term treatment. Plasma exchange (PE) could be combined in severe patients, immunosuppressant could be used for refractory patients or for recurrence and using an anti-epileptic drug to control seizures may benefit cognition.

**Conclusions:** Short-term memory loss, FBDS, psychiatric symptoms, and hyponatremia were key features in identifying anti-LGI1 encephalitis. Serum and CSF antibody tests should be considered in diagnosis criteria. Steroids with IVIG should be recommended, PE was combined for use in severe patients, immunosuppressant therapy might improve outcomes if recurrence or progression occurred, and control seizures might benefit cognition. The useful ways to reduce relapse rate were early identification, clear diagnosis, rapid treatment, and maintaining long-term treatment. The follow-up advice was suggested according to the research of paraneoplastic syndrome, and concern about tumors was vital as well.

## Introduction

Anti-leucine-rich glioma inactivated 1 (LGI1) encephalitis is an autoimmune encephalitis (AE), whose clinical presentations are memory disturbances, faciobrachial dystonic seizures (FBDS), confusion or psychiatric disorders, and hyponatremia (1, 2). Anti-LGI1 encephalitis can be diagnosed through clinical features, magnetic resonance imaging (MRI), serum or cerebrospinal fluid (CSF) tests, and electroencephalogram (EEG) (3). The gold standard for diagnosis is a positive LGI1 antibody in serum or CSF. Most articles about clinical presentations and treatment programs of anti-LGI1 encephalitis are case reports or case series, thus, overall understanding and an especially comprehensive treatment program of the disease are needed. As a result, the main objective of this study is to analyze clinical features and therapeutic effects of anti-LGI1 encephalitis by reviewing relevant literature systematically.

## Methods

This systematic review was conducted according to the Preferred Reporting Items for Systematic reviews and Meta-Analyses (PRISMA) guidelines (4).

### Criteria for Considering Studies for Review

Studies were included with the following designs: case reports/series, case–control studies, cross-sectional studies, cohort studies, or clinical trials, if available. Studies reporting clinical features and/or treatment programs, involving patients diagnosed with confirmed anti-LGI1 encephalitis according to clinical criteria and presence of antibodies in serum and/or CSF were included. There was no restriction on age, sex, ethnicity of patients, or year of publication in this review. Other types of articles such as short communications, animal studies, unavailable full-text articles, and articles not published in Chinese or English were excluded.

### Search Strategy

We searched PubMed, EMBASE, and the Cochrane Library for literature published in Chinese or English up until April 2021. General and MeSH search terms were “LGI1 protein, human (Supplementary Concept) AND encephalitis (MeSH).” Up to date articles were traced for supplementary searching.

We assessed the titles and abstracts of identified records based on the screening criteria above. Studies meeting the inclusion criteria were retrieved as full-text articles and subjected to predefined eligibility criteria.

### Data Extraction and Analysis

Data were independently extracted by two authors. Demographic figures of characteristics, clinical presentation, neuroimaging, serum and CSF analysis findings, descriptive findings in the EEG, treatment programs, therapeutic effects, and other clinical information of subjects in each study were extracted. Categorical variables were summarized by counts and percentages, while continuous variables were pooled by median and range.

## Results

### Included Studies

**Figure 1:**
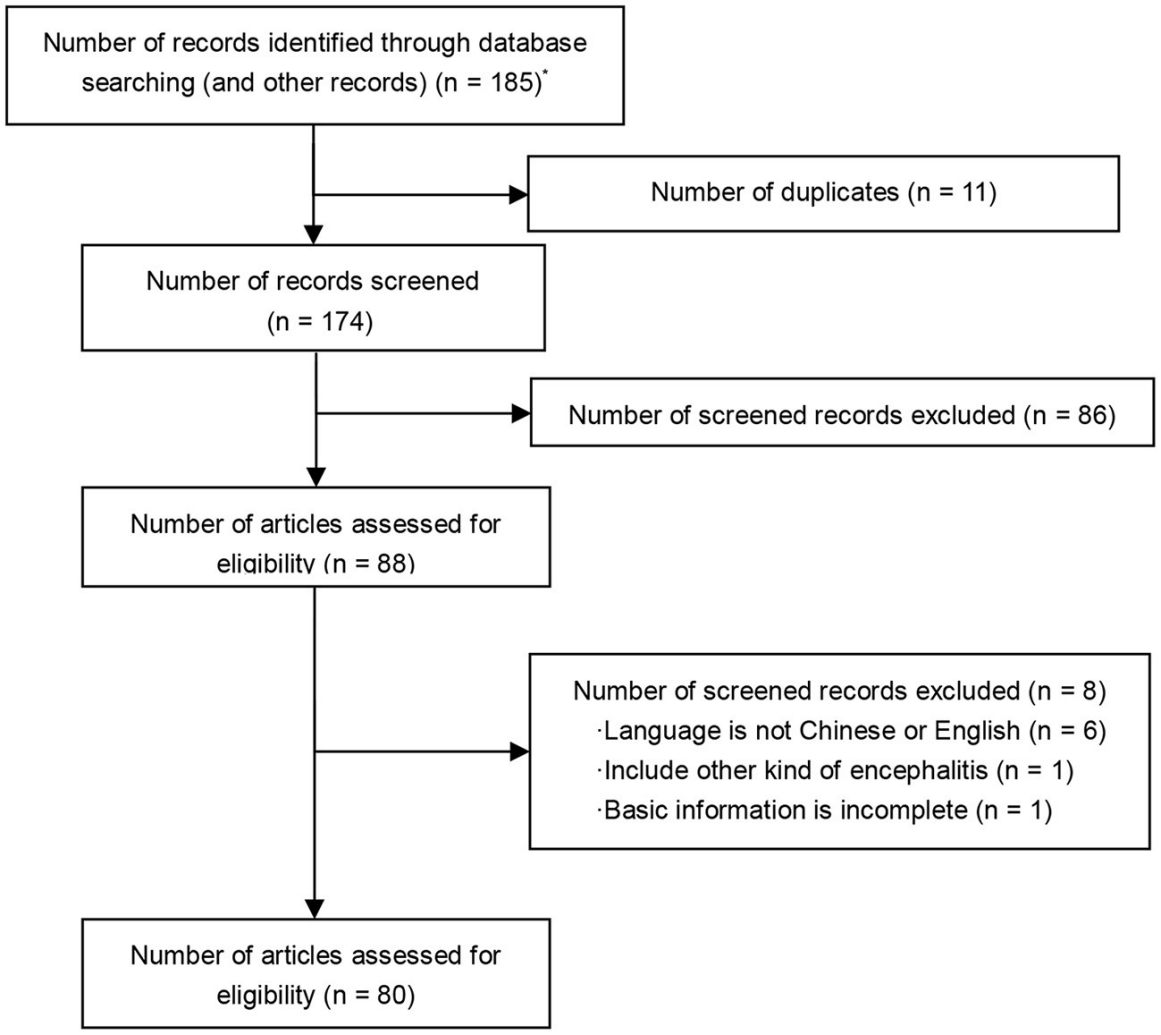
PRISMA flow diagram. *A total of 185 articles were identified through database searching: PubMed (*n* = 155), The Cochrane Library (*n* = 7), EMBASE (*n* = 22), supplementary reference (*n* = 1).

We identified 185 articles from the initial search. After removal of 11 duplications, 87 out of 174 articles met the inclusion criteria. A total of 80 articles were eligible for the review, consisting of 65 case reports and 15 case series (Figure 1).

### Population Characteristics

A total of 485 cases with confirmed positive LGI1 antibody in serum and/or CSF were included. The demographic and clinical information of the included cases are summarized in Table 1.

**Table 1:** Clinical features.

**References**	**No**.	**Age/** gender^*a^	**Cognition**		Psychiatric symptoms^*c^	**Confusion**	**Seizures**		**Sleep disturbances**	Other symptoms^*^^d^	Other diseases^*^^e^
			**Memory**	Others^*b^			**FBDS**	**Others**			
Park et al. (5)	1	43/F	+	–	–	–	+	–	–	–	N
Kuehn et al. (6)	2	64/M	–	L	–	–	+	–	–	S, P	N
Perez et al. (7)	3	70/M	–	L	–	–	–	+	–	M, H	N
Zangrandi et al. (8)	4	74/F	+	L, V, E	Dp, E, A, Ap, H, Dl, AE	–	+	–	+	U, D	D, H, dyslipidemia, prostatic hypertrophy
Hye (9)	5	72/M	+	O, L	E	–	–	–	–	M	N
Rahangdale et al. (10)	6	38/F	–	–	E	–	+	+	–	M, S	Migraine headache
Sato et al. (11)	7	59/M	+	O, A, C	H, A	–	+	–	–	–	N
Ji et al. (12)	8	67/M	+	–	H, Dl, E	+	+	+	+	U	H
Ibrahim et al. (13)	9	33/F	+	L, V	–	–	+	+	–	M, S, A	N
Shen et al. (14)	10	41/F	+	–	–	–	+	+	–	F, P, A	N
Chapelet et al. (15)	11	75/F	+	L, E	–	–	+	+	–	–	ACTH-dependent hypercortisolism
AlHakeem et al. (16)	12	7/F	–	–	Ag	–	–	+	+	F, Iu F	N
Yuan et al. (17)	13	60/M	+	O, V, L	H, E, An	–	+	+	–	–	Essential thrombocythemia
Tu et al. (18)	14	43/F	+	O	H, E	–	+	+	+	–	N
Zouras et al. (19)	15	69/M	–	–	–	+	–	+	+	S, P, W	N
Li et al. (20)	16	56/M	+	–	–	–	–	–	–	F	N
Zhao et al. (21)	17	46/M	+	–	–	–	–	–	–	–	N
	18	75/F	–	–	–	–	–	–	–	M	N
	19	41/F	–	–	–	–	–	–	–	S	N
Haitao et al. (22)	20	64/M	–	–	–	+	–	–	+	UT	H, V
	21	44/M	+	–	–	+	–	–	–	–	V
Cooper et al. (23)	22	79/M	–	–	–	–	–	+	+	–	H, hyperlipidemia
Cash et al. (24)	23	75/M	+	L, A, E	H, Dp, An, E	–	–	+	+	S, D, Fa, A	H, lumbar disk disease, pseudogout, decreased hearing
Attwood et al. (25)	24	83/F	+	A	–	–	+	+	–	S	oral squamous cell carcinoma, locally advanced breast cancer
Frattini et al. (26)	25	75/M		+	Ap	–	–	–	+	M, Fa, H, C, W	N
Renard et al. (27)	26	80/M	+	–	–	–	–	+	+	W	N
Sweeney et al. (28)	27	68/M	–	–	–	–	–	+	–	–	H, hypothyroidism, dyslipidemia, previous cardiac arrest presented
Tumminelli et al. (29)	28	78/M	–	–	H	–	–	+	+	S	D
Ahn et al. (30)	29	72/M	–	L	–	+	+	+	–	–	lung cancer
Takahashi et al. (31)	30	41/F	+	–	–	–	–	–	–	P	N
Pollak and Moran (32)	31	57/M	+	–	H, Dl, Dp, An, Ag	–	+	+	+	–	degenerative lumbar canal stenosis
Naasan et al. (33)	32	53/M	+	L	H, An	–	+	–	–	Fa, D, UT	N
	33	64/M	+	L, E	An, Dl	–	–	+	–	D	N
	34	55/F	+	–	H, Ap, An	+	+	+	–	–	N
Miao et al. (34)	35	39/F	–	–	–	–	–	+	–	A	N
Fidzinski et al. (35)	36	92/F	–	–	–	–	–	+	–	–	N
d'Orsi et al. (36)	37	68/M	+	L	H	–	–	+	–	–	N
Dubey et al. (37)	38	70/M	+	–	Dp, H	–	–	–	–	W	prostate cancer
	39	66/M	+	A, V	–	+	–	+	–	–	N
Gong et al. (38)	40	59/F	+	–	–	–	+	+	+	–	N
Peter-Derex et al. (39)	41	65/-	+	–	–	–	–	–	+	–	N
Kurtis et al. (40)	42	74/M	–	O, L, A	H	+	–	+	+	S, M	D, right hand congenital malformation, atrial fibrillation
Tofaris et al. (41)	43	77/M	+	O, E, L	Ap	–	–	–	–	M	N
	44	60/M	+	–	Ap	–	+	–	–	M	N
Casault et al. (42)	45	65/M	+	L	Ag	+	–	+	–	M	D
Rachdi et al. (43)	46	66/M	–	–	–	–	–	+	–	M	Crohn's disease
Mir et al. (44)	47	7/F	–	–	Ap	–	+	+	–	–	N
Schultze-Amberger et al. (45)	48	80/F	+	–	–	–	+	–	–	–	D, H, cardiac and renal insufficiency, chronic bronchopulmonary disease
Wang et al. (46)	49	18/M	+	–	Ap, E, A	–	–	+	–	–	N
Steriade et al. (47)	50	18/M	–	–	H, Ap	+	–	–	–	M, P, A	N
Kaymakamzade et al. (48)	51	31/M	–	–	–	–	–	–	–	F, P, A	N
Zhao and Yang (49)	52	59/F		+	–	–	–	+	–	A	N
	53	82/F	+	L	–	+	–	+	–	Iu	Brain atrophy
Messelmani et al. (50)	54	59/M	+	O	H, A	–	+	+	–	–	V, D, verruca seborrhoica, gastroesophageal reflux disease, intraductal papillary mucinous neoplasia of the pancreas
Schimmel et al. (51)	55	14/M	+	–	Dp, A	–	–	–	–	–	D
Brown et al. (52)	56	68/F	+	–	Dl	–	–	+	–	–	N
Nilsson and Blaabjerg (53)	57	67/F	+	–	–	+	–	+	–	–	N
Szots et al. (54)	58	50/M	+	L	An, Ag	–	+	–	–	–	N
	59	48/M	+	–	H	–	–	+	–	–	N
Agazzi et al. (55)	60	67/M	+	O	E	–	–	+	+	–	N
Sen et al. (56)	61	62/F	–	–	–	–	–	+	–	–	N
Wang et al. (57)	62	30/F	+	O, C	–	–	–	–	–	–	N
Vogrig et al. (58)	63	54/F	+	E	–	–	–	+	–	–	N
Fantaneanu et al. (59)	64	57/M	+	L	–	–	–	+	–	–	N
Yelam et al. (60)	65	47/M	–	–	A	–	–	+	–	–	Hepatitis B
Beimer and Selwa (61)	66	51/F	+	L	–	–	–	+	+	–	Asthma, hypothyroidism
Espinosa-Jovel et al. (62)	67	56/M	–	–	–	–	–	–	–	–	N
Rizzi et al. (63)	68	55/M	+	L, E	Dp, H	+	+	+	+	–	N
Bing–Lei et al. (64)	69	50/F	+	–	–	–	+	–	–	–	N
	70	45/F	+	U	–	–	+	–	–	–	N
	71	64/M	+	L	–	–	–	+	–	–	N
Hor et al. (65)	72	69/M	+	–	–	+	–	–	–	–	Nephrotic syndrome, thymoma
Krastinova et al. (66)	73	72/M	+	–	Dp	+	–	+	–	–	H, glaucoma
Gravier Dumonceau et al. (67)	74	76/F	+	L	–	–	–	+	–	–	N
Zheng et al. (68)	75	76/F	+	–	H	–	–	+	–	–	N
Incecik et al. (69)	76	8/F	–	–	H, A	+	+	–	–	–	N
Li et al. (70)	77	47/M		–	N/A	N/A	+	–	N/A	N/A	N
	78	78/M		–	N/A	N/A	+	–	N/A	N/A	Small cell lung cancer
	79	58/F		+	N/A	N/A	–	+	N/A	N/A	N
	80	64/M		–	N/A	N/A	+	+	N/A	N/A	N
	81	48/F		+	N/A	N/A	–	+	N/A	N/A	N
	82	72/M		–	N/A	N/A	+	–	N/A	N/A	N
	83	34/F		+	N/A	N/A	–	+	N/A	N/A	N
	84	65/M		+	N/A	N/A	+	+	N/A	N/A	N
	85	39/F		–	N/A	N/A	+	+	N/A	N/A	N
	86	77/M		–	N/A	N/A	+	–	N/A	N/A	N
Li et al. (71)	87	64/M	+	–	–	N/A	+	+	+	A	N
	88	69/M	+	O	H	N/A	+	+	+	M, A	N
	89	60/F	+	O	H, E, Ap	N/A	–	+	+	A	N
	90	63/F	+	O	H	N/A	+	+	–	–	N
	91	67/M	+	–	H	N/A	–	–	+	–	N
	92	73/M	+	O	Ap	N/A	+	–	–	–	N
	93	41/F	+	–	An, E, Dl	N/A	+	–	–	–	N
	94	70/M	+	–	An	N/A	+	–	–	–	N
Gao et al. (72)	95	55/M	+	N/A	A	–	+	+	+	N/A	N
	96	50/F	+	N/A	–	–	+	+	–	N/A	N
	97	27/F	+	N/A	–	+	+	+	–	N/A	N
	98	41/F	+	N/A	–	+	+	+	–	N/A	N
	99	43/M	–	N/A	–	–	+	+	–	N/A	N
	100	62/M	+	N/A	–	–	+	+	–	N/A	N
	101	33/M	+	N/A	–	+	+	+	–	N/A	N
	102	72/M	+	N/A	–	–	+	+	–	N/A	N
	103	57/M	+	N/A	–	+	+	+	–	N/A	N
	104	75/M	+	N/A	–	–	+	+	+	N/A	N
Wang et al. (73)	105	22/M	+	N/A	Dl, A		+	+	–	–	N
	106	66/M	+	N/A	Ap		+	+	–	F	N
	107	24/F	+	N/A	H, Ap		+	+	–	F, P	N
	108	18/F	+	N/A	Dl, Ap		+	+	–	–	N
	109	21/F	+	N/A	H, Dl, A		+	–	–	F	Psychiatric disease
	110	36/F	+	N/A	H, Dl, Ap		+	+	–	–	N
	111	40/F	+	N/A	Ap		+	+	–	–	N
	112	50/F	+	N/A	H, A		+	+	–	–	N
	113	61/M	+	N/A	A		+	+	–	–	N
	114	43/F	+	N/A	Dl, Ap, A		+	+	–	–	N
	115	63/M	+	N/A	H, Dl, A		+	+	–	–	N
	116	38/F	+	N/A	H, Dl, A		+	+	–	–	N
	117	44/M	+	N/A	Dl, Ap		+	+	–	F, D	N
Aurangzeb et al. (74)	118	71/M	N/A	N/A	N/A	N/A	–	+	N/A	N/A	N
	119	67/M	N/A	N/A	N/A	N/A	+	+	N/A	N/A	N
	120	61/M	N/A	N/A	N/A	N/A	+	–	N/A	N/A	N
	121	78/M	N/A	N/A	N/A	N/A	+	+	N/A	N/A	N
	122	92/M	N/A	N/A	N/A	N/A	+	–	N/A	N/A	N
	123	63/M	N/A	N/A	N/A	N/A	–	+	N/A	N/A	N
	124	69/M	N/A	N/A	N/A	N/A	+	–	N/A	N/A	N
	125	56/M	N/A	N/A	N/A	N/A	+	+	N/A	N/A	N
	126	68/M	N/A	N/A	N/A	N/A	+	–	N/A	N/A	N
	127	76/M	N/A	N/A	N/A	N/A	+	+	N/A	N/A	N
	128	64/F	N/A	N/A	N/A	N/A	+	+	N/A	N/A	N
	129	63/F	N/A	N/A	N/A	N/A	+	+	N/A	N/A	N
	130	69/M	N/A	N/A	N/A	N/A	+	+	N/A	N/A	N
	131	66/M	N/A	N/A	N/A	N/A	+	–	N/A	N/A	N
	132	64/F	N/A	N/A	N/A	N/A	+	+	N/A	N/A	N
	133	53/M	N/A	N/A	N/A	N/A	+	+	N/A	N/A	N
Yu et al. (75)	134	41/F	N/A	N/A	+	N/A	+	+	–	S	N
	135	46/M	N/A	N/A	–	N/A	–	–	–	–	N
	136	75/F	N/A	N/A	+	N/A	+	–	–	–	N
	137	54/M	N/A	N/A	–	N/A	–	+	–	–	N
Shin et al. (76)	138	43/F	+	N/A	A	+	+	+	–	–	N
	139	43/M	–	N/A	–	–	–	+	–	–	N
	140	61/M	+	N/A	A	–	+	–	–	–	N
	141	70/F	+	N/A	–	+	+	–	–	–	N
	142	73/M	+	N/A	Dp	+	+	–	+	M, C, Iu, A	N
	143	41/M	–	N/A	–	–	–	+	–	–	N
	144	60/F	–	N/A	A	+	–	+	–	–	N
	145	61/F	+	N/A	–	–	+	+	–	–	N
	146	78/F	–	N/A	A, E	+	+	–	+	M, A	N
	147	66/M	–	N/A	–	–	+	+	–	–	N
	148	53/M	+	N/A	–	+	+	+	–	–	Renal cell carcinoma
	149	62/F	+	N/A	–	–	–	+	–	–	N
	150	55/M	+	N/A	–	+	+	+	+	C, Iu	N
	151	58/M	+	N/A	–	–	+	+	–	–	N
van Sonderen et al. (77)	152	64 (31–84)/ M 25, F 13	37/38	O 17/33	34/38 (Ap 18, Di 14, Ag 13, A 10)	N/A	18/38	25/38	20/31	PN 5/32, 3/34, W 9/33	Tumor 4/38
Ariño et al. (78)	153	61 (32–80)/ M 50, F 26	76/76	N/A	A 33/76, E 49/76, M 23/76	N/A		67/76	33/76	N/A	Tumor 5/16
Celicanin et al. (79)	154	62 (29–84)/ M 9, F 7	16/16	N/A	PC 4/16, E 4/16, H 4/16, An 3/16, Dl 2/16	N/A	4/16	12/16	2/16	P 3/16, A 4/16	N
Li et al. (80)	155	58 (23–82)/ M 15, F 4	13/19	N/A	14/19	N/A	13/19	14/19	–	M, S	4D, 1 schizophrenia, 1 cerebral infarction
Yang et al. (81)	156	56.9 (37–73)/ M 20, F 4	18/24	N/A	PC or H 8/24	N/A	9/24	18/24	N/A	N/A	N
Zhang et al. (82)	157	46.6 (37–54)/ M 6, F 3		7/9	5/9	N/A	3/9	5/9	N/A	N/A	N
Lai et al. (83)	158	60 (30–80)/ M 37, F 20	57/57	N/A	N/A	N/A		42/51	N/A	N/A	Tumor 6/57
Bastiaansen et al. (84)	159	66 (49–82)/ M 29, F 13, 53 N/A	42/95	L5, V 28, E 30	N/A	N/A	32/42			24/42	Tumor 3/42

*N/A, not applicable; FBDS, faciobrachial dystonic seizures;*

*a *M, male; F, female*;

*^b^ *L, language ability deficits; V, impaired visuospatial ability; E, impaired executive function; O, impaired orientation; A, inattention; C, calculation disturbance; U, decreased comprehension*;

*c *H, hallucinations; E, emotional stability; A, aberrant motor behavior; AE, appetite/eating changes; Ap, apathy; Ag, agitation; An, anxiety; Dl, delusions; Dp, depression; PC, personality change; Di, disinhibition; M, mental disorder*;

* *d, P, pain; S, sensory symptoms; M, motor symptoms; H, hard to swallow; F, fever; U, unconscious; A, autonomic symptoms; W, weight loss; D, dizziness; Iu, urinary incontinence; Iuf, urinary and fecal incontinence; UT, uncharacteristic tear dropping; Fa, fatigue; C, constipation; PN, peripheral nervous system symptoms*;

* *e, N, no tumor; H, hypothyroidism; D, diabetes; V, vitiligo*.

There were 281/431 (65.20%) men, 150/431 (34.80%) women, and 54 patients with unknown gender. Age ranged from 7 to 92 years (mean age 59.61 years), including four pediatric patients (44, 51, 69, 78). Fifty-three participants were not included due to unclear demographic details.

### Clinical Features

The main clinical features in anti-LGI1 encephalitis are summarized in Table 2. A total of 412 out of 485 cases showed cognitive impairments. Apart from 21 patients, 464 reported certain categories, in which 349 (75.22%) had short-term memory loss, 30 (17.96%) had impaired orientation, 27 (16.17%) had language ability deficits, 37 (22.16%) had impaired executive function, 5 (2.99%) had inattention, 32 (19.16%) had impaired visuospatial ability, 2 (1.20%) had calculation disturbance, and 1 (0.60%) had decreased comprehension.

**Table 2:** Main clinical features in anti-LGI1 encephalitis.

	**Total**
	***n/N*** **(%)^a^**
Short-term memory loss	349/464 (75.22%)
Psychiatric symptoms	124/215 (57.67%)
FBDS	135/257 (52.53%)
Other seizures excluding FBDS	176/257 (68.48%)
Sleep disturbances	106/309 (34.30%)
Confusion	27/100 (27.00%)
Hyponatremia	196/357 (54.90%)
Hyper intensity in the medial temporal lobe or hippocampus in MRI (T2 / FLAIR)	279/380 (73.42%)
High metabolism in the medial temporal lobe or hippocampus in PET	30/43 (69.77%)
Positive rate of anti-LGI1 in serum	244/252 (96.83%)
Positive rate of anti-LGI1 in CSF	171/221 (77.38%)
Positive rate of anti-LGI1 in both serum and CSF	139/197 (70.56%)

*FBDS, faciobrachial dystonic seizures; CSF, cerebrospinal fluid*.

a *Data reported as n/N (%), where N is the total number of patients with details applicable for each feature and n is the number of patients presenting features*.

There were 307 patients who reported the occurrence of psychiatric symptoms. Overall, 124/215 (57.67%) were abnormal [two articles (78, 79) which lacked the total number of patients with psychiatric symptoms were excluded]. Apart from 21/30 cases, 277 reported the classification of psychiatric symptoms. A total of 77 (27.80%) had emotionalist ability deficits, 62 (22.38%) had aberrant motor behaviors, 35 (12.64%) had apathy, 29 (10.47%) had hallucinations, 23 (8.30%) had mental disorders, 17 (6.14%) had agitation, 16 (5.78%) had delusions, 14 (5.05%) had disinhibition, 12 (4.33%) had anxiety, 8 (2.89%) had depression, 8 (2.89%) had personality changes or hallucinations, 4 (1.44%) had personality changes, and 1 (0.36%) had appetite/eating changes.

Seizures were also reported. In total, 27/100 (27.00%) had confusion, 135/257 (52.53%) had FBDS, and 176/257 (68.48%) had other seizures. Other symptoms, such as sleep disturbances (106/309, 34.30%), autonomic symptoms (16/169, 9.47%), motor symptoms (15/169, 8.88%), weight loss (13/164, 7.93%), fever (8/169, 4.73%), peripheral nervous system symptoms (5/163, 3.07%), dizziness (5/169, 2.96%) were reported as well.

### Combined Diseases

For comorbidities, 24/430 (5.58%) reported tumor incidence, 3/430 (0.70%) reported vitiligo, and there were other comorbidities reported as well, such as diabetes, hypothyroidism, dyslipidemia, hypothyroidism, etc.

### Laboratory Examination

Data of neuroimaging, assay systems for the LGI1 antibody test, EEG, and treatments are summarized in Supplementary Table 1. The therapeutic effects and other clinical information are summarized in Supplementary Table 2.

For laboratory examination, 196/357 (54.90%) reported hyponatremia. For antibody detection, 241/249 (96.78%) reported anti-LGI1 in serum, while 171/221 (77.38%) reported anti-LGI1 in CSF. There were also other antibodies reported, such as VGKC (19/76, 25.00%), NMDAR (2/76, 2.63%), CASPR2 (1/76, 1.32%), and AMPAR (1/76, 1.32%).

### Auxiliary Examinations

For neuroimaging, 279/380 (73.42%) reported hyper intensity in the medial temporal lobe or hippocampus in MRI (T2/FLAIR), while 30/43 (69.77%) reported high metabolism in the medial temporal lobe or hippocampus in PET. Of 288 cases with EEG outcomes, 101 (35.07%) reported epileptiform discharge, comparatively, 100 (34.72%) reported abnormalities but no epileptiform discharge in EEGs, and the other 87 (30.21%) reported no abnormal EEGs. After comparing the syndrome of seizures and the results of EEG in 126 cases, 26 (20.63%) FBDS and 31 (24.60%) other seizures showed epileptiform discharge in EEG, 30 (23.81%) FBDS and 35 (27.78%) other seizures showed abnormalities but no epileptiform discharge in EEG, and 24 (19.05%) FBDS and 22 (17.46%) other seizures showed no abnormal EEGs.

### Treatments and Outcomes

Treatments and outcomes in anti-LGI1 encephalitis are summarized in Table 3. Among the 390 cases, 358 documented the processes of treatment. As a result, 285/358 (79.61%) received steroids, and 106/285 (37.19%) received steroid pulse therapy. Aside from 38 cases which reported on the combination of intravenous immunoglobulin (IVIG) and plasma exchange (PE), 166/320 (51.88%) received IVIG, and 12 cases received this treatment more than once. For other treatments, 20/320 (6.25%) received PE, and 47/358 (13.13%) received immunosuppressants including rituximab (17/47), azathioprine (15/47), mycophenolate mofetil (8/47), cyclophosphamide (6/47), tacrolimus (2/47), and cyclosporine (1/47). For anti-epileptic treatment, 122 of 390 cases recorded the use of anti-epileptic drugs, and 86/122 (55.74%) received drug therapy, in which 26 cases reported the reactions but only 5/26 (19.23%) reported that it helped. Combined therapy from cases with details of treatment is summarized in Supplementary Table 3.

**Table 3:** Treatments and outcomes in anti-LGI1 encephalitis.

	**Total**	**Complete remission**	**Remission**	**Relapsed**
	***n/N*** **(%)^a^**	***n/N*** **(%)^b^**	***n/N*** **(%)^b^**	***n/N*** **(%)^b^**
Steroids only	97/241 (40.25%)	22/43 (51.16%)	18/43 (41.86%)	9/43 (20.93%)
IVIG only	38/241 (15.77%)	9/16 (56.25%)	5/16 (31.25%)	1/16 (6.25%)
Steroids and IVIG	100/241 (41.49%)	25/60 (41.67%)	33/60 (55.00%)	5/60 (8.33%)
PE*	20/319 (6.27%)	4/12 (33.33%)	6/12 (50.00%)	5/12 (41.67%)
Immunosuppressants*	47/358 (13.13%)	5/13 (38.46%)	8/13 (61.54%)	5/13 (38.46%)
Total	358/390 (91.79%)	142/295 (48.14%)	111/295 (37.63%)	35/295 (11.86%)

*IVIG, intravenous immunoglobulin; PE, Plasma exchange*.

a *Data reported as n/N (%), where N is the total number of patients with details of each therapy and n is the number of patients who received the mentioned therapy*.

b *Data reported as n/N (%), where N is the total number of patients analyzed in the initial visit of each therapy and n is the number of patients in different outcomes who received each therapy in the initial visit*.

PE^*^, PE was not used alone according to our results. The combined therapy included PE with steroids, PE with steroids and IVIG, PE with steroids and immunosuppressants, and PE with steroids, IVIG, and immunosuppressants. Immunosuppressants^*^: Immunosuppressants were not used alone according to our results. The combined therapy included immunosuppressants with steroids, immunosuppressants with steroids and IVIG, immunosuppressants with steroids and PE, and immunosuppressants with steroids, IVIG, and PE.

Overall, 295 of 390 cases reported outcomes of treatments. A total of 137/295 (46.44%) achieved complete remission, 109/295 (36.95%) achieved remission, 46/295 (15.59%) relapsed, 14/295 (4.75%) did not reach remission, 1/295 (0.34%) rejected further treatment, and 15/295 (5.08%) died.

For the initial visit, 241 of 390 cases kept detailed records of combined therapy, and 100/241 (41.49%) received both steroids and IVIG. Among the cases reported on outcomes, 25/60 (41.67%) who received a combination of steroids and IVIG achieved complete remission, 33/60 (55.00%) achieved remission, 2/60 (3.33%) did not achieve remission, and 5/60 (8.33%) relapsed. Comparatively, in 97/241 (40.25%) cases receiving steroids only, 22/43 (51.16%) achieved complete remission, 18/43 (41.86%) achieved remission, 3/43 (6.98%) did not achieve remission, and 9/43 (20.93%) relapsed among the recorded cases. In total, 38/241 (15.77%) received IVIG only, and it turned out that 9/16 (56.25%) achieved complete remission, 5/16 (31.25%) achieved remission, 1/16 (6.25%) did not achieve remission, 1/16 (6.25%) rejected further treatment, and 1/16 (6.25%) relapsed. Overall, 17/269 (6.32%) used PE, and 4/12 (33.33%) achieved complete remission, 6/12 (50.00%) achieved remission, 2/12 (16.67%) did not achieve remission, and 5/12 (41.67%) relapsed.

For the visit after recurrence, 35 of 215 cases relapsed, of which 6/35 (17.14%) did not use first-line treatment, and 21 (60.00%) did not maintain long-term treatment. A total of 15 of 35 cases kept detailed records of therapy, of which 10/15 (66.67%) used steroids, 5/15 (33.33%) used IVIG, 3/15 (20.00%) used PE, 7/15 (46.67%) used immunosuppressants, and 2/15 (13.33%) were not treated (Table 4). All the above 12 cases achieved remission or complete remission in the end, but 3 patients died, possibly attributed to leukemia, myocardial infarction, and unknown causes.

**Table 4:** Treatment and outcomes after relapse.

**Treatment**	**Complete remission**	**Remission**	**Death**	**Total**
Steroids only	0	1	1	2
IVIG only	2	0	0	2
Steroids and IVIG	0	2	0	2
Immunosuppressants	0	1	0	1
Steroids and immunosuppressants	2	0	0	2
Steroids, IVIG, and immunosuppressants	1	0	0	1
Steroids, PE, and immunosuppressants	1	2	0	3
None	0	0	2	2
Total	6	6	3	15
Steroids	4 (40.00%)	5 (50.00%)	1 (10.00%)	10
IVIG	3 (60.00%)	2 (40.00%)	0	5
PE	1 (33.33%)	2 (66.67%)	0	3
Immunosuppressants	4 (57.14%)	3 (42.85%)	0	7
None	0 (0.00%)	0 (0.00%)	2 (100.00%)	2

*IVIG, intravenous immunoglobulin; PE, Plasma exchange*.

## Discussion

This review described clinical features and therapeutic effects of anti-LGI1 encephalitis comprehensively. According to our results, the most common symptom of anti-LGI1 encephalitis was short-term memory loss, which is a common characteristic in other AE (84). A quarter of patients with anti-LGI1 encephalitis suffered from cognitive decline in orientation, while fewer patients had impairment in visuospatial skills and executive function. Contrary to our results, Bastiaansen et al. (84) discovered that patients with anti-LGI1 encephalitis showed similarities in frequency and severity of visuospatial and executive function impairment as those with anti-GABA_B_R encephalitis (~70% in anti-LGI1 encephalitis and 55% in anti-GABA_B_R encephalitis). We drew the controversial conclusion that this was possibly because some cases we included did not contain complete information on cognitive disorders, which could serve as a reminder for clinicians to pay more attention to cognitive impairments in patients with anti-LGI1 encephalitis.

The frequency occurrence of other seizure types was higher than FBDS, likely due to the fact that too many case reports were included in our study. According to previous research (74), FBDS was considered as pathognomonic for anti-LGI1 encephalitis, in which EEG typically showed prominent muscle artifacts (lasting 0.5–1.6 s). Meanwhile, FBDS was also reported to be the most common seizure type in anti-LGI1 encephalitis, as well as a distinction among anti-LGI1 encephalitis and other AE (84).

As AE can affect any brain network involving initiating and regulating sleep, all types of sleep disorders can occur, with distinct association, frequency, and intensity (85). Compared to other research (84), the rate of sleep disorders in anti-LGI1 encephalitis was lower based on our results, thus it reminded us to pay more attention to patients' sleep problems especially for clinicians.

A multiple-center study (86) demonstrated that in 379 patients, anti-NMDAR-AE patients had the highest incidence of tumors, accounting for 8.79% from analysis. As a kind of AE, anti-LGI1 encephalitis might be associated with paraneoplastic syndrome (PNS). According to previous case series, PNS has a 0–31% chance of revealing tumors (77–83, 87), among which thymoma and lung cancer were considered the most common ones (1). Nonetheless, 5.58% of our included cases showed carcinogenesis, including oral squamous cell carcinoma and locally advanced lung cancer (30, 70), breast cancer (25), prostate cancer (37), thymoma (65), renal cell carcinoma (76), etc., which are inconsistent with the former results. It is likely the tumor types mentioned above were not included, so further investigations are needed to gather more complete information. As the lack of a specific suggestion of tumor screening for AE, the tumor screening routine of PNS should provide a valuable reference (88), suggesting a repeated second screening after 3–6 months, followed by regular screening every 6 months for 4 years if the initial screening is negative in patients with PNS. For immune disorder in anti-LGI1 encephalitis and PNS, the incidence rate of malignant tumors seems to be significantly higher in anti-LGI1 encephalitis patients (89). According to the follow-up regulation in PNS, subsequent specialty consultations are suggested in anti-LGI1 encephalitis regardless of negative tumor markers or imaging examinations.

In our study, hyponatremia was regarded as the most common electrolyte disturbance. Muhr et al. (90) concluded that the underlying mechanisms leading to hyponatremia might be inadequate ADH secretion. Additionally, severe hyponatremia could be regarded as a precursor of anti-LGI1 encephalitis.

In our study, the positive rate of LGI1 antibodies in CSF was 77.38%, similar to a cohort study (with a positive rate of 78%). The positive rate in serum was 96.83%, suggesting a higher sensitivity in diagnosing anti-LGI1 encephalitis. Despite the relatively lower positive rate of LGI1 antibodies in CSF, there were still advantages in distinguishing different forms of encephalitis from CSF antibody tests.

As for neuroimaging, our results showed that MRI (T2/FLAIR) and PET both had a relatively high positive rate in diagnosis. Additionally, according to a meta-analysis (91), the detection sensitivity of PET in anti-LGI1 encephalitis was 87% (79–92%), *I*^2^ of 0% (*p* = 0.89), suggesting that PET, as a new medical technology, was of high value in diagnosis of anti-LGI1 encephalitis. As for EEG, epileptiform discharge and abnormal EEG with no epileptiform discharge were two key features, in line with another study (92).

Interestingly, three patients from two articles (22, 50) reported vitiligo, as the authors hypothesized that vitiligo might work as an inducer for anti-LGI1 encephalitis.

As anti-LGI1 encephalitis was mostly reported in adults (44), we did a comparison of symptoms for three anti-LGI1 encephalitis pediatric patients, and found all patients had psychiatric symptoms and different types of seizures, but none had cognitive disturbance (16, 44, 69), highlighting the necessity to be suspicious of AE when new onset seizures and psychiatric symptoms occur in children.

First-line immunotherapy of AE included corticosteroids, IVIG, and PE (93). According to our research, steroids (93.02%, 40/43), IVIG (87.50%, 14/16), and combined use (96.67%, 58/60) all had a high remission rate. However, in some previous studies, the remission rate of using steroids alone was 100% (1/1, 4/4) (70, 71), using IVIG alone was 87.5% (7/8) (72), and combined using was 100% (8/8, 4/4) (70, 71). These differences may be due to the increase in the representativeness of the population after the expansion of the sample size. Among 189 cases with follow-up over 6 months, only 1 case (43) reported adverse events after using steroids. Accepting steroid intravenous impulse therapy was considered relatively safe. In a recent retrospective study (94), the combined treatment with PE and IVIG was found to be more effective than IVIG alone. Steroids combined with IVIG was reported to have good responses and few adverse events. So, more research on the efficacy of other combined therapies in relapsed patients or those with bad responses are needed in the future.

Since recovery and symptom remission were accompanied by a decline of antibody titers in other AE (95), it was hypothesized that aiming to get a decrease of LGI1 antibody titers might be a primary therapy approach. PE and immunoadsorption (IA) both provide an opportunity for the extracorporeal elimination of circulating antibodies (96). Zhang *et al*. (97) demonstrated that therapeutic PE might be an effective rescue therapy for rapid functional improvement in patients with severe steroid/IVIG refractory antibody-associated AE, including anti-LGI1 encephalitis, and with no fatal adverse events. Another pilot study (96) with 21 AE cases including 4 anti-LGI1 encephalitis cases illustrated that both IA and PE resulted in a moderate to marked clinical improvement, also with a relatively low adverse event risk. As a result, on account of its high cost and invasive damage, PE might be a suitable therapy for emergent treatment in critically ill patients to achieve more rapid remission. Due to the limited numbers of anti-LGI1 encephalitis patients included, more research is needed to further test the safety and long-term efficacy of PE. Additionally, since PE can only eliminate antibodies that already exist rather than intervening in their production, how to combine PE with another therapy to prevent recurrence and achieve complete remission should also be taken into consideration.

Second-line immunotherapy of AE included rituximab and cyclophosphamide (93), and was suggested to be immediately started in those who failed to respond or deteriorated during first-line immunotherapy (98). Nepal et al. (99) found rituximab was effective for treatment of AE with an acceptable toxicity profile, while Lee et al. (100) found that high doses of rituximab showed benefits in refractory AE patients. The international consensus (101) recommended rituximab for cases refractory to the first-line agent in both anti-NMDAR AE children and adults, while cyclophosphamide was suggested 1–3 months after second-line initiation. As the cases included in these three studies above were mostly anti-NMDAR AE, the results did not exactly match our research. We found that, after adding immunosuppressants, 100% of relapsed patients reached remission (42.85%) or complete remission (57.14%), among which rituximab alone had efficacy against anti-LGI1 encephalitis. Despite the relatively high rate of remission, adding rituximab also led to the occurrence of adverse incidence such as infusion-related reactions (IRRs) (15.7%), pneumonia (6.0%) and severe sepsis (1.1%), which we cannot afford to neglect.

A systematic review including 87 anti-NMDAR AE children showed that only 7% of patients relapsed on mycophenolate mofetil, azathioprine, or methotrexate (102), though there was little evidence supporting their importance in refractory or relapsed anti-LGI1 encephalitis. Our research found that 7 out of 15 cases had used these 4 agents after relapse, and all of them achieved complete remission or remission afterwards.

Cognition might be related to FBDS. Thompson et al. (103) found that FBDS showed significant time-sensitive responses to immunotherapy, and the development of cognitive impairment could be prevented with their surcease. Overall, 10% showed cessation of FBDS with anti-epileptic drugs alone, while 51% showed cessation of FBDS 30 days after addition of first-line immunotherapy. Our result showed that only 19.23% of epilepsy symptoms were controlled after using anti-epileptic drugs. The choice to use anti-epileptic drugs depends on the physicians' assessment, and the efficacy needs further research.

Inadequate dosage and duration of first-line agents were possibly responsible for recurrence (77). Our results showed that 17.14% of relapsed patients did not initiate first-line treatment, and 60.00% did not maintain treatment. So, in order to prevent relapse, early recognition, definite diagnosis, rapid treatment, and first-line treatment with adequate dosage and duration are all necessary in the process.

In view of the fact that our included studies were mostly case reports, this systematic review has a number of limitations, such as increased risks of reporting and selection biases. The integrity of clinical features, test results, and treatment effects from included articles might limit the conclusions as well. And the lack of follow-up details affected the final judgment of therapeutic effects. Though there is not any result from the randomized controlled trial, the result of this study could be referred. We are looking forward to more high-quality studies about efficacy and safety of anti-LGI1 encephalitis treatment.

## Conclusion

In this review, according to our results, it is suggested that clinicians should suspect or consider anti-LGI1 encephalitis when the following symptoms appear: short-term memory loss, psychiatric symptoms, hyponatremia, seizures, or FBDS, especially in patients aged over 40. Brain MRI scanning and serum and CSF antibody tests should be done when considering diagnosis. EEG is necessary when suspicious seizures occur, and using anti-epileptic drugs to control seizures may benefit cognition. As for treatment, the statistics of our study suggest the combination of steroids with IVIG at the onset; gradually decreasing oral steroids and regular follow-up afterwards are also necessary. If anti-LGI1 encephalitis becomes severe, PE could be introduced. If anti-LGI1 encephalitis is refractory or recurs, immunosuppressant therapy such as rituximab, cyclophosphamide, mycophenolate mofetil, azathioprine, and methotrexate may provide potential benefits. Due to the high risk of incidence rate of malignant tumors in the population of anti-LGI1 encephalitis, a follow-up advice reference to PNS is suggested, which requires a repeated second screening after 3–6 months, followed by regular screening every 6 months for 4 years.

## Data Availability Statement

The original contributions presented in the study are included in the article/Supplementary Material, further inquiries can be directed to the corresponding author/s.

## Author Contributions

JT, JS, MW, and TL contributed to conceive and design this systematic review. YT and TL conducted the study selection and extracted the data from the selected articles. YT ran the data analysis. YT, ZY, and MS drafted the manuscript with supervision from TL and JN.

## Funding

This work was supported by the Fundamental Research Funds for the Central Universities (No. 2019-JYB-TD-007) and Qihuang Scholar Foundation (China).

## Conflict of Interest

The authors declare that the research was conducted in the absence of any commercial or financial relationships that could be construed as a potential conflict of interest.

## Publisher's Note

All claims expressed in this article are solely those of the authors and do not necessarily represent those of their affiliated organizations, or those of the publisher, the editors and the reviewers. Any product that may be evaluated in this article, or claim that may be made by its manufacturer, is not guaranteed or endorsed by the publisher.
