# Supplementary material for: Clinical Features and Therapeutic Effects of Anti-leucine-rich Glioma Inactivated 1 Encephalitis: A Systematic Review
Source: Front Neurol. 2022 Jan 12;12:791014. doi: 10.3389/fneur.2021.791014 (PMC8791026; doi:10.3389/fneur.2021.791014)
Supplement: Supplementary file 1 [file Data_Sheet_1.docx]

Supplementary Material

# Supplementary Tables

**Supplementary Table 1. Laboratory examination and assay systems for LGI-1 antibody in cases.**

| No. | Hyponatremia | MRI*^a^ | PET*^b^ | EEG*^c^ | Assay system for anti-LGI1 | anti-LGI1 | | Other antibodies | Anti-epileptic treatment | Curative effect |
| --- | --- | --- | --- | --- | --- | --- | --- | --- | --- | --- |
|  |  |  |  |  |  | serum | CSF |  |  |  |
| 1 | - | + | + | A | N/A | + | N/A | - | N/A | N/A |
| 2 | 127 | + | + | A | N/A | + | + | - | N/A | + |
| 3 | - | + | + | N/A | N/A | + | N/A | - | N/A | N/A |
| 4 | - | - | - | E | IF | + | N/A | VGKC | + | - |
| 5 | + | N/A | + | A | N/A | N/A | + | VGKC | - | N/A |
| 6 | - | - | N/A | N/A | N/A | + | N/A | - | + | - |
| 7 | 130 | + | N/A | E | N/A | + | N/A | VGKC | + | - |
| 8 | 120 | + | N/A | N/A | CBA | + | + | MNDAR | + | - |
| 9 | - | + | + | E | IF | + | N/A | VGKC | + | - |
| 10 | 132 | + | N/A | E | IIF | + | + | - | + | N/A |
| 11 | 119 | - | N/A | A | N/A | N/A | + | - | + | N/A |
| 12 | - | N/A | N/A | A | N/A | + | N/A | - | - | N/A |
| 13 | 120.1 | + | N/A | E | N/A | + | + | - | + | N/A |
| 14 | + | - | N/A | A | N/A | N/A | + | - | + | N/A |
| 15 | 126 | + | N/A | N | N/A | + | N/A | VGKC | + | N/A |
| 16 | 126.1 | + | - | N/A | N/A | + | + | - | - | N/A |
| 17 | - | + | N/A | N/A | IIF | + | - | - | - | N/A |
| 18 | - | - | N/A | A | IIF | + | + | - | + | N/A |
| 19 | - | - | N/A | N/A | IIF | + | + | - | - | N/A |
| 20 | - | - | - | E | N/A | + | + | NMDAR, CASPR2, AMPAR | - | N/A |
| 21 | - | + | + | N/A | N/A | + | + | - | - | N/A |
| 22 | 127 | - | N/A | N/A | N/A | + | N/A | VGKC | - | N/A |
| 23 | + | + | + | A | N/A | + | N/A | - | + | - |
| 24 | 128 | - | N/A | A | N/A | + | + | - | + | N/A |
| 25 | + | + | - | N | N/A | + | N/A | - | - | N/A |
| 26 | - | + | + | A | N/A | + | N/A | VGKC | + | - |
| 27 | - | + | N/A | A | N/A | + | N/A | VGKC | + | - |
| 28 | - | - | N/A | N | IIF | + | N/A | - | - | N/A |
| 29 | 130 | - | N/A | E | N/A | + | N/A | - | + | N/A |
| 30 | - | + | N/A | N/A | N/A | + | N/A | - | + | N/A |
| 31 | + | + | N/A | N/A | N/A | + | N/A | VGKC | + | - |
| 32 | - | N/A | N/A | N/A | N/A | + | N/A | VGKC | - | N/A |
| 33 | + | + | N/A | N/A | N/A | N/A | N/A | VGKC | - | N/A |
| 34 | - | + | N/A | N/A | N/A | N/A | N/A | - | - | N/A |
| 35 | - | + | N/A | E | IIF | + | + | - | N/A | N/A |
| 36 | - | - | - | N | CBA | + | N/A | - | + | N/A |
| 37 | 127 | + | - | E | N/A | + | + | - | + | - |
| 38 | 127 | - | N/A | N | CBA | + | N/A | VGKC | - | N/A |
| 39 | 125 | + | N/A | E | N/A | + | N/A | VGKC | - | - |
| 40 | - | + | + | N | N/A | + | + | - | + | + |
| 41 | - | + | N/A | A | IF | N/A | + | - | - | N/A |
| 42 | - | N/A | N/A | E | CBA | + | + | VGKC | - | N/A |
| 43 | 118 | N/A | N/A | A | N/A | + | N/A | VGKC | - | N/A |
| 44 | 125 | + | N/A | A | N/A | + | N/A | VGKC | - | N/A |
| 45 | + | + | + | N | N/A | N/A | + | - | - | N/A |
| 46 | - | - | N/A | N | N/A | + | - | - | + | - |
| 47 | 133 | - | N/A | E | N/A | + | + | VGKC, GAD65 | + | - |
| 48 | + | + | N/A | A | N/A | + | + | - | + | - |
| 49 | 126.5 | + | N/A | A | N/A | + | - | - | + | - |
| 50 | - | - | - | N | CBA | + | N/A | - | - | N/A |
| 51 | 132 | + | N/A | N/A | N/A | + | + | - | - | N/A |
| 52 | - | + | N/A | E | N/A | + | + | - | + | N/A |
| 53 | 122 | + | N/A | N/A | N/A | + | N/A | - | + | - |
| 54 | 116 | - | N/A | A | N/A | + | N/A | VGKC | + | - |
| 55 | - | + | N/A | N | CBA | + | - | - | - | N/A |
| 56 | 130 | + | + | E | CBA | + | N/A | VGKC | + | N/A |
| 57 | + | + | N/A | A | CBA | + | + | - | - | N/A |
| 58 | 126 | + | N/A | E | N/A | + | N/A | - | + | + |
| 59 | + | - | N/A | E | N/A | + | - | - | + | + |
| 60 | 125 | + | N/A | N/A | N/A | + | + | - | + | N/A |
| 61 | - | + | N/A | N | N/A | + | N/A | - | - | N/A |
| 62 | - | + | N/A | N/A | N/A | + | + | - | - | N/A |
| 63 | + | N/A | N/A | A | N/A | + | + | - | + | N/A |
| 64 | - | + | N/A | N | N/A | N/A | + | - | + | N/A |
| 65 | - | + | N/A | A | N/A | + | N/A | - | + | - |
| 66 | - | N/A | N/A | A | N/A | + | N/A | - | + | - |
| 67 | - | + | + | N/A | N/A | N/A | + | - | N/A | N/A |
| 68 | - | + | N/A | A | N/A | + | N/A | - | + | - |
| 69 | 135.8 | + | N/A | N | IIF | + | - | - | + | N/A |
| 70 | - | N/A | N/A | A | IIF | + | + | - | + | N/A |
| 71 | 132 | + | N/A | A | IIF | + | - | - | + | N/A |
| 72 | - | + | N/A | A | N/A | + | - | - | - | N/A |
| 73 | - | + | N/A | N/A | N/A | + | + | - | - | N/A |
| 74 | - | + | - | N/A | N/A | + | N/A | - | + | + |
| 75 | 124 | N/A | - | A | IIF | + | - | - | + | - |
| 76 | 135 | + | N/A | E | N/A | + | N/A | - | - | N/A |
| 77 | - | N/A | N/A | N | N/A | + | - | N/A | + | N/A |
| 78 | 130 | N/A | N/A | A | N/A | + | + | N/A | + | N/A |
| 79 | - | + | N/A | A | N/A | + | + | N/A | + | N/A |
| 80 | 132 | N/A | N/A | E | N/A | + | + | N/A | + | N/A |
| 81 | - | N/A | N/A | A | N/A | + | + | N/A | + | N/A |
| 82 | 133 | N/A | N/A | N | N/A | + | - | N/A | + | N/A |
| 83 | 115 | + | N/A | E | N/A | + | + | N/A | + | N/A |
| 84 | 132 | N/A | N/A | A | N/A | + | + | N/A | + | N/A |
| 85 | - | N/A | N/A | E | N/A | + | + | N/A | + | N/A |
| 86 | - | N/A | N/A | N | N/A | + | - | N/A | + | N/A |
| 87 | - | + | N/A | N | IIF | + | + | N/A | + | N/A |
| 88 | 130 | N/A | N/A | N | IIF | + | - | N/A | + | N/A |
| 89 | 127.9 | + | N/A | N | IIF | + | - | N/A | + | N/A |
| 90 | 126 | N/A | N/A | A | IIF | + | + | N/A | + | N/A |
| 91 | 131.4 | + | N/A | E | IIF | + | + | N/A | + | N/A |
| 92 | 121.2 | N/A | N/A | N | IIF | + | + | N/A | + | N/A |
| 93 | 132 | N/A | N/A | N | IIF | + | - | N/A | + | N/A |
| 94 | - | N/A | N/A | N | IIF | + | - | N/A | + | N/A |
| 95 | 129 | N/A | + | E | IIF | + | + | N/A | + | N/A |
| 96 | - | + | N/A | A | IIF | + | + | N/A | + | N/A |
| 97 | 134 | + | N/A | A | IIF | + | - | N/A | + | N/A |
| 98 | 134 | + | - | E | IIF | + | + | N/A | + | N/A |
| 99 | 121 | N/A | N/A | E | IIF | + | + | N/A | + | N/A |
| 100 | 123 | + | N/A | A | IIF | + | + | N/A | + | N/A |
| 101 | - | + | N/A | A | IIF | + | + | N/A | + | N/A |
| 102 | 130 | N/A | N/A | A | IIF | + | + | N/A | + | N/A |
| 103 | 134 | + | + | A | IIF | + | + | N/A | + | N/A |
| 104 | 110 | N/A | N/A | A | IIF | + | + | N/A | + | N/A |
| 105 | - | - | N/A | A | IIF | - | + | N/A | N/A | N/A |
| 106 | - | - | N/A | A | IIF | - | + | N/A | N/A | N/A |
| 107 | - | - | N/A | N | IIF | + | - | N/A | N/A | N/A |
| 108 | - | + | N/A | N | IIF | + | - | N/A | N/A | N/A |
| 109 | - | - | N/A | N | IIF | + | - | N/A | N/A | N/A |
| 110 | - | + | N/A | A | IIF | + | - | N/A | N/A | N/A |
| 111 | - | + | N/A | A | IIF | - | + | N/A | N/A | N/A |
| 112 | - | - | N/A | N | IIF | + | + | N/A | N/A | N/A |
| 113 | - | - | N/A | A | IIF | - | + | N/A | N/A | N/A |
| 114 | - | - | N/A | N | IIF | + | + | N/A | N/A | N/A |
| 115 | - | - | N/A | A | IIF | + | - | N/A | N/A | N/A |
| 116 | - | - | N/A | A | IIF | + | - | N/A | N/A | N/A |
| 117 | - | - | N/A | A | IIF | + | - | N/A | N/A | N/A |
| 118 | N/A | N/A | N/A | E | N/A | positive in serum or CSF | | N/A | N/A | N/A |
| 119 | N/A | N/A | N/A | N | N/A | positive in serum or CSF | | N/A | N/A | N/A |
| 120 | N/A | N/A | N/A | N | N/A | positive in serum or CSF | | N/A | N/A | N/A |
| 121 | N/A | N/A | N/A | E | N/A | positive in serum or CSF | | N/A | N/A | N/A |
| 122 | N/A | N/A | N/A | E | N/A | positive in serum or CSF | | N/A | N/A | N/A |
| 123 | N/A | N/A | N/A | N | N/A | positive in serum or CSF | | N/A | N/A | N/A |
| 124 | N/A | N/A | N/A | N | N/A | positive in serum or CSF | | N/A | N/A | N/A |
| 125 | N/A | N/A | N/A | E | N/A | positive in serum or CSF | | N/A | N/A | N/A |
| 126 | N/A | N/A | N/A | N | N/A | positive in serum or CSF | | N/A | N/A | N/A |
| 127 | N/A | N/A | N/A | N | N/A | positive in serum or CSF | | N/A | N/A | N/A |
| 128 | N/A | N/A | N/A | E | N/A | positive in serum or CSF | | N/A | N/A | N/A |
| 129 | N/A | N/A | N/A | E | N/A | positive in serum or CSF | | N/A | N/A | N/A |
| 130 | N/A | N/A | N/A | E | N/A | positive in serum or CSF | | N/A | N/A | N/A |
| 131 | N/A | N/A | N/A | N | N/A | positive in serum or CSF | | N/A | N/A | N/A |
| 132 | N/A | N/A | N/A | E | N/A | positive in serum or CSF | | N/A | N/A | N/A |
| 133 | N/A | N/A | N/A | N | N/A | positive in serum or CSF | | N/A | N/A | N/A |
| 134 | 133.8 | + | N/A | N/A | IIF | + | + | N/A | + | N/A |
| 135 | 134 | + | N/A | N/A | IIF | + | - | N/A | - | N/A |
| 136 | 124 | - | N/A | N/A | IIF | + | - | N/A | + | N/A |
| 137 | 123 | + | N/A | N/A | IIF | + | + | N/A | + | N/A |
| 138 | - | + | + | E | IIF | positive in serum or CSF | | N/A | N/A | N/A |
| 139 | - | + | + | E | IIF | positive in serum or CSF | | N/A | N/A | N/A |
| 140 | - | + | + | A | IIF | positive in serum or CSF | | N/A | N/A | N/A |
| 141 | - | + | + | E | IIF | positive in serum or CSF | | N/A | N/A | N/A |
| 142 | - | - | - | E | IIF | positive in serum or CSF | | N/A | N/A | N/A |
| 143 | - | + | + | E | IIF | positive in serum or CSF | | N/A | N/A | N/A |
| 144 | - | + | N/A | N | IIF | positive in serum or CSF | | N/A | N/A | N/A |
| 145 | - | + | N/A | N/A | IIF | positive in serum or CSF | | N/A | N/A | N/A |
| 146 | - | + | N/A | A | IIF | positive in serum or CSF | | N/A | N/A | N/A |
| 147 | - | - | + | N | IIF | positive in serum or CSF | | N/A | N/A | N/A |
| 148 | - | - | + | N | IIF | positive in serum or CSF | | N/A | N/A | N/A |
| 149 | - | + | N/A | E | IIF | positive in serum or CSF | | N/A | N/A | N/A |
| 150 | - | + | + | E | IIF | positive in serum or CSF | | N/A | N/A | N/A |
| 151 | - | - | + | E | IIF | positive in serum or CSF | | N/A | N/A | N/A |
| 152 | 24/37 | 26/35 | N/A | N 16, A 9, E 11 | IMH &  CBA | 38/38 | 9/17 | N/A | N/A | N/A |
| 153 | 52/70 | 73/76 | N/A | N/A | IMH &  CBA | 47/51 | 51/51 | N/A | N/A | N/A |
| 154 | 9/16 | 11/16 | 7/9 | N 2, A 3, E 9 | IIF | 16/16 | 7/16 | N/A | N/A | N/A |
| 155 | 5/19 | 3/19 | N/A | N 6, A 6, E 5 | IIF | N/A | 19/19 | N/A | 13/19 | N/A |
| 156 | 14/24 | 17/24 | N/A | A 11, E 12 | IIF &  CBA | 24/24 | 20/24 | N/A | + | N/A |
| 157 | 2/9 | 6/9 | N/A | N/A | IIF | 9/9 | 6/9 | N/A | N/A | N/A |
| 158 | 28/47 | 43/51 | N/A | N 8, A 11, E 15 | CBA | 57/57 | | N/A | N/A | N/A |
| 159 | N/A | 24/41 | N/A | N 17/38, A 12/38, E 9/38 | CBA | positive in serum or CSF | | N/A | N/A | N/A |

Abbreviations: N/A: not applicable. IVIG: Intravenous immunoglobulin; PE: Plasma exchange. *a: +: hyper intensity in medial temporal lobe or hippocampus. *b: +: high metabolism in medial temporal lobe or hippocampus. *c: N: Normal. A: Abnormalities but no epileptiform discharge in EEGs. E: Epileptiform discharge. IF: Immunofluorescence test. IIF: indirect immunofluorescence test. CBA: cell-based assays. IMH: Immunohistochemistry.

**Supplementary Table 2. Treatment, outcomes and follow up in cases.**

| No. | Initial Visit | | | | | | | Follow up*^g^ | After recurrence/ Need to change treatment plan | | | | | | Follow up*^g^ |
| --- | --- | --- | --- | --- | --- | --- | --- | --- | --- | --- | --- | --- | --- | --- | --- |
|  | Steroids | Intravenous impulse therapy*^d^ | IVIG | PE | Immunosuppressant*^e^ | Prognosis*^f^ | Relapse (n) |  | Steroids | Intravenous impulse therapy*^d^ | IVIG | PE | Immunosuppressant*^e^ | Prognosis*^f^ |  |
| 1 | N/A | N/A | N/A | N/A | N/A | CR | N/A | N/A | N/A | N/A | N/A | N/A | N/A | N/A | N/A |
| 2 | N/A | N/A | N/A | N/A | N/A | N/A | N/A | N/A | N/A | N/A | N/A | N/A | N/A | N/A | N/A |
| 3 | N/A | N/A | N/A | N/A | N/A | D | N/A | N/A | N/A | N/A | N/A | N/A | N/A | N/A | N/A |
| 4 | + | - | Once | + | - | R | N/A | - | N/A | N/A | N/A | N/A | N/A | N/A | N/A |
| 5 | + | + | More | - | M, R | R | 0 | 6 m | N/A | N/A | N/A | N/A | N/A | N/A | N/A |
| 6 | + | + | More | - | - | CR | 0 | 12 m | N/A | N/A | N/A | N/A | N/A | N/A | N/A |
| 7 | + | + | - | - | - | R | 0 | 24 m | N/A | N/A | N/A | N/A | N/A | N/A | N/A |
| 8 | + | + | - | - | - | R | 0 | 12 m | N/A | N/A | N/A | N/A | N/A | N/A | N/A |
| 9 | + | + | More | - | - | R | 0 | 7 m | N/A | N/A | N/A | N/A | N/A | N/A | N/A |
| 10 | + | + | More | - | - | CR | 0 | 3 m | N/A | N/A | N/A | N/A | N/A | N/A | N/A |
| 11 | + | + | Once | - | A | CR | 0 | 9 m | N/A | N/A | N/A | N/A | N/A | N/A | N/A |
| 12 | + | + | Once | - | - | CR | N/A | - | N/A | N/A | N/A | N/A | N/A | N/A | N/A |
| 13 | + | + | Once | - | - | R | 1 | 12 m | - | - | + | - | M | R | - |
| 14 | + | + | - | - | - | CR | 0 | 3 m | N/A | N/A | N/A | N/A | N/A | N/A | N/A |
| 15 | + | - | More | - | - | N | N/A | N/A | + | + | - | + | - | CR | 36m |
| 16 | + | N/A | Once | - | - | CR | 0 | 1 m | N/A | N/A | N/A | N/A | N/A | N/A | N/A |
| 17 | + | + | Once | - | - | CR | 0 | 20 m | N/A | N/A | N/A | N/A | N/A | N/A | N/A |
| 18 | + | + | Once | - | - | R | 1 | 5 m | N/A | N/A | N/A | N/A | N/A | D | N/A |
| 19 | + | - | Once | - | - | CR | 0 | 22 m | N/A | N/A | N/A | N/A | N/A | N/A | N/A |
| 20 | + | - | Once | - | - | CR | 0 | 18 m | N/A | N/A | N/A | N/A | N/A | N/A | N/A |
| 21 | + | - | - | - | - | R | 1 | 2 m | + | - | + | - | - | R | 24m |
| 22 | + | + | - | - | - | CR | 0 | 12 m | N/A | N/A | N/A | N/A | N/A | N/A | N/A |
| 23 | + | + | Once | - | R | R | N/A | - | N/A | N/A | N/A | N/A | N/A | N/A | N/A |
| 24 | + | + | - | + | - | R | 1 | 9 w | + | - | - | + | M | R | - |
| 25 | + | + | - | - | - | R | 1 | - | N/A | N/A | N/A | N/A | N/A | D | N/A |
| 26 | + | - | - | + | - | N | 0 | 5 w | + | - | + | - | M, A | CR | 8m |
| 27 | + | + | - | + | M | CR | 1 | 4 m | + | - | - | - | M | CR | 12m |
| 28 | + | + | - | - | - | CR | 0 | 2 m | N/A | N/A | N/A | N/A | N/A | N/A | N/A |
| 29 | + | + | - | - | - | CR | N/A | - | N/A | N/A | N/A | N/A | N/A | N/A | N/A |
| 30 | + | + | - | - | - | CR | 0 | 96 m | N/A | N/A | N/A | N/A | N/A | N/A | N/A |
| 31 | + | + | Once | + | - | R | 1 | 6 m | + | + | - | + | C | R | - |
| 32 | + | + | - | - | - | CR | 0 | 2 m | - | - | + | - | A | CR | - |
| 33 | + | + | - | - | - | R | 1 | 3 w | + | + | + | - | R | R | 18m |
| 34 | - | - | - | - | - | R | 1 | - | + | + | - | - | - | R | 24m |
| 35 | N/A | N/A | N/A | N/A | N/A | N/A | N/A | N/A | N/A | N/A | N/A | N/A | N/A | N/A | N/A |
| 36 | - | - | More | - | - | CR | 0 | 25 m | N/A | N/A | N/A | N/A | N/A | N/A | N/A |
| 37 | - | + | - | - | - | R | 0 | 24 m | N/A | N/A | N/A | N/A | N/A | N/A | N/A |
| 38 | + | + | - | + | - | R | 0 | 18 m | N/A | N/A | N/A | N/A | N/A | N/A | N/A |
| 39 | + | + | - | + | M | CR | 0 | 4 m | N/A | N/A | N/A | N/A | N/A | N/A | N/A |
| 40 | - | - | Once | - | - | CR | N/A | - | N/A | N/A | N/A | N/A | N/A | N/A | N/A |
| 41 | + | - | More | - | - | R | N/A | - | N/A | N/A | N/A | N/A | N/A | N/A | N/A |
| 42 | + | - | - | - | A | R | 1 | 6 w | + | + | - | - | - | D | 18 m |
| 43 | + | - | Once | - | - | R | 0 | 6 w | N/A | N/A | N/A | N/A | N/A | N/A | N/A |
| 44 | + | - | + | - | - | R | 0 | 24 m | N/A | N/A | N/A | N/A | N/A | N/A | N/A |
| 45 | + | + | + | - | A | R | 1 | 1 m | + | + | + | - | - | R | N/A |
| 46 | + | + | More | - | - | CR | 0 | 6 m | Adverse events | - | + | - | C | CR | 12 m |
| 47 | + | + | More | - | - | CR | 0 | 3 m | N/A | N/A | N/A | N/A | N/A | N/A | N/A |
| 48 | N/A | N/A | N/A | N/A | N/A | D | N/A | N/A | N/A | N/A | N/A | N/A | N/A | N/A | N/A |
| 49 | + | + | - | - | - | CR | N/A | - | N/A | N/A | N/A | N/A | N/A | N/A | N/A |
| 50 | + | + | - | - | - | CR | 0 | 9 m | N/A | N/A | N/A | N/A | N/A | N/A | N/A |
| 51 | + | + | - | + | - | N | 0 | 1 m | + | + | - | - | - | CR | 12m |
| 52 | + | + | - | - | - | CR | 0 | 24 m | N/A | N/A | N/A | N/A | N/A | N/A | N/A |
| 53 | + | N/A | - | - | - | N | 0 | 12 m | N/A | N/A | N/A | N/A | N/A | N/A | N/A |
| 54 | - | - | - | - | - | CR | 0 | 18 m | N/A | N/A | N/A | N/A | N/A | N/A | N/A |
| 55 | + | + | - | + | - | R | 0 | 60 m | - | - | - | - | M | N/A | N/A |
| 56 | + | - | - | + | - | CR | 1 | 2 m | + | - | - | + | R | CR | 15 m |
| 57 | + | + | - | + | - | CR | 0 | 1 m | N/A | N/A | N/A | N/A | N/A | N/A | N/A |
| 58 | N/A | N/A | N/A | N/A | N/A | R | N/A | - | N/A | N/A | N/A | N/A | N/A | N/A | N/A |
| 59 | N/A | N/A | N/A | N/A | N/A | CR | N/A | - | N/A | N/A | N/A | N/A | N/A | N/A | N/A |
| 60 | + | + | - | - | - | CR | 1 | 48 m | + | + | - | - | A | CR | 120 m |
| 61 | + | + | - | - | - | CR | 0 | 18 m | N/A | N/A | N/A | N/A | N/A | N/A | N/A |
| 62 | + | N/A | + | - | - | CR | N/A | - | N/A | N/A | N/A | N/A | N/A | N/A | N/A |
| 63 | + | - | More | - | - | R | 0 | 30 m | N/A | N/A | N/A | N/A | N/A | N/A | N/A |
| 64 | + | + | Once | - | R | R | N/A | - | N/A | N/A | N/A | N/A | N/A | N/A | N/A |
| 65 | + | + | Once | - | - | R | 0 | 2 m | N/A | N/A | N/A | N/A | N/A | N/A | N/A |
| 66 | + | + | - | - | - | R | 0 | 1 w | N/A | N/A | N/A | N/A | N/A | N/A | N/A |
| 67 | N/A | N/A | N/A | N/A | N/A | CR | N/A | - | N/A | N/A | N/A | N/A | N/A | N/A | N/A |
| 68 | N/A | N/A | N/A | N/A | N/A | D | N/A | N/A | N/A | N/A | N/A | N/A | N/A | N/A | N/A |
| 69 | + | + | - | - | - | R | 0 | 3 m | N/A | N/A | N/A | N/A | N/A | N/A | N/A |
| 70 | + | - | Once | - | - | R | 0 | 3 m | N/A | N/A | N/A | N/A | N/A | N/A | N/A |
| 71 | + | - | Once | - | - | R | 0 | 3 m | N/A | N/A | N/A | N/A | N/A | N/A | N/A |
| 72 | + | - | Once | - | - | R | 0 | 12 m | + | - | + | - | - | CR | 21 m |
| 73 | + | + | More | - | - | CR | 0 | 3 m | N/A | N/A | N/A | N/A | N/A | N/A | N/A |
| 74 | + | + | Once | - | R, C | R | 0 | 1 m | - | - | - | - | C | CR | - |
| 75 | N/A | N/A | + | N/A | N/A | R | 0 | 1 m | N/A | N/A | N/A | N/A | N/A | N/A | N/A |
| 76 | + | + | Once | - | - | CR | N/A | - | N/A | N/A | N/A | N/A | N/A | N/A | N/A |
| 77 | + | - | + | - | - | CR | 0 | 6–16 (10.2 ± 3.2) m | N/A | N/A | N/A | N/A | N/A | N/A | N/A |
| 78 | - | N/A | + | - | - | Refusal of further treatment | N/A |  | N/A | N/A | N/A | N/A | N/A | N/A | N/A |
| 79 | + | - | - | - | - | CR | 0 |  | N/A | N/A | N/A | N/A | N/A | N/A | N/A |
| 80 | + | - | + | - | - | CR | 0 |  | N/A | N/A | N/A | N/A | N/A | N/A | N/A |
| 81 | + | - | + | - | - | R | 0 |  | N/A | N/A | N/A | N/A | N/A | N/A | N/A |
| 82 | + | - | + | - | - | CR | 0 |  | N/A | N/A | N/A | N/A | N/A | N/A | N/A |
| 83 | + | - | + | - | - | CR | 0 |  | N/A | N/A | N/A | N/A | N/A | N/A | N/A |
| 84 | + | - | + | - | - | CR | 0 |  | N/A | N/A | N/A | N/A | N/A | N/A | N/A |
| 85 | + | - | + | - | - | CR | 0 |  | N/A | N/A | N/A | N/A | N/A | N/A | N/A |
| 86 | + | - | + | - | - | CR | 0 |  | N/A | N/A | N/A | N/A | N/A | N/A | N/A |
| 87 | + | + | + | - | - | R | 0 | 3 m | N/A | N/A | N/A | N/A | N/A | N/A | N/A |
| 88 | + | + | - | - | - | R | 0 | 3 m | N/A | N/A | N/A | N/A | N/A | N/A | N/A |
| 89 | + | + | - | - | - | R | 0 | 3 m | N/A | N/A | N/A | N/A | N/A | N/A | N/A |
| 90 | + | + | + | - | - | R | 0 | 3 m | N/A | N/A | N/A | N/A | N/A | N/A | N/A |
| 91 | + | + | + | - | - | R | 0 | 3 m | N/A | N/A | N/A | N/A | N/A | N/A | N/A |
| 92 | + | + | + | - | - | R | 0 | 3 m | N/A | N/A | N/A | N/A | N/A | N/A | N/A |
| 93 | + | + | - | - | - | CR | 0 | 3 m | N/A | N/A | N/A | N/A | N/A | N/A | N/A |
| 94 | + | + | - | - | - | R | 0 | 3 m | N/A | N/A | N/A | N/A | N/A | N/A | N/A |
| 95 | - | N/A | + | - | - | CR | 1 | 18 m | - | - | + | - | - | CR | 30 m |
| 96 | - | N/A | + | - | - | CR | 0 | 24 m | N/A | N/A | N/A | N/A | N/A | N/A | N/A |
| 97 | - | N/A | + | - | - | CR | 0 | 15 m | N/A | N/A | N/A | N/A | N/A | N/A | N/A |
| 98 | - | N/A | + | - | - | CR | 0 | 14 m | N/A | N/A | N/A | N/A | N/A | N/A | N/A |
| 99 | - | N/A | + | - | - | R | 0 | 13 m | N/A | N/A | N/A | N/A | N/A | N/A | N/A |
| 100 | - | N/A | + | - | - | CR | 0 | 13 m | N/A | N/A | N/A | N/A | N/A | N/A | N/A |
| 101 | - | N/A | - | - | - | CR | 1 | 10 m | - | - | + | - | - | CR | 13 m |
| 102 | - | N/A | + | - | - | R | 0 | 10 m | N/A | N/A | N/A | N/A | N/A | N/A | N/A |
| 103 | - | N/A | - | - | - | CR | 0 | 10 m | N/A | N/A | N/A | N/A | N/A | N/A | N/A |
| 104 | - | N/A | + | - | - | N | 0 | 2 m | - | - | + | - | - | D | N/A |
| 105 | + | N/A | - | - | - | R | 0 | 2 m | N/A | N/A | N/A | N/A | N/A | N/A | N/A |
| 106 | - | N/A | + | - | - | R | 0 | 2 m | N/A | N/A | N/A | N/A | N/A | N/A | N/A |
| 107 | + | N/A | + | - | - | R | 0 | 2 m | N/A | N/A | N/A | N/A | N/A | N/A | N/A |
| 108 | + | N/A | - | - | - | R | 0 | 2 m | N/A | N/A | N/A | N/A | N/A | N/A | N/A |
| 109 | + | N/A | - | - | - | R | 0 | 2 m | N/A | N/A | N/A | N/A | N/A | N/A | N/A |
| 110 | - | N/A | + | - | - | CR | 0 | 2 m | N/A | N/A | N/A | N/A | N/A | N/A | N/A |
| 111 | + | N/A | - | - | - | R | 0 | 2 m | N/A | N/A | N/A | N/A | N/A | N/A | N/A |
| 112 | - | N/A | + | - | - | CR | 0 | 2 m | N/A | N/A | N/A | N/A | N/A | N/A | N/A |
| 113 | + | N/A | + | - | - | R | 0 | 2 m | N/A | N/A | N/A | N/A | N/A | N/A | N/A |
| 114 | - | N/A | + | - | - | R | 0 | 2 m | N/A | N/A | N/A | N/A | N/A | N/A | N/A |
| 115 | + | N/A | + | - | - | R | 0 | 2 m | N/A | N/A | N/A | N/A | N/A | N/A | N/A |
| 116 | + | N/A | + | - | - | R | 0 | 2 m | N/A | N/A | N/A | N/A | N/A | N/A | N/A |
| 117 | + | N/A | - | - | - | CR | 0 | 2 m | N/A | N/A | N/A | N/A | N/A | N/A | N/A |
| 118 | N/A | N/A | N/A | N/A | N/A | N/A | N/A | N/A | N/A | N/A | N/A | N/A | N/A | N/A | N/A |
| 119 | N/A | N/A | N/A | N/A | N/A | N/A | N/A | N/A | N/A | N/A | N/A | N/A | N/A | N/A | N/A |
| 120 | N/A | N/A | N/A | N/A | N/A | N/A | N/A | N/A | N/A | N/A | N/A | N/A | N/A | N/A | N/A |
| 121 | N/A | N/A | N/A | N/A | N/A | N/A | N/A | N/A | N/A | N/A | N/A | N/A | N/A | N/A | N/A |
| 122 | N/A | N/A | N/A | N/A | N/A | N/A | N/A | N/A | N/A | N/A | N/A | N/A | N/A | N/A | N/A |
| 123 | N/A | N/A | N/A | N/A | N/A | N/A | N/A | N/A | N/A | N/A | N/A | N/A | N/A | N/A | N/A |
| 124 | N/A | N/A | N/A | N/A | N/A | N/A | N/A | N/A | N/A | N/A | N/A | N/A | N/A | N/A | N/A |
| 125 | N/A | N/A | N/A | N/A | N/A | N/A | N/A | N/A | N/A | N/A | N/A | N/A | N/A | N/A | N/A |
| 126 | N/A | N/A | N/A | N/A | N/A | N/A | N/A | N/A | N/A | N/A | N/A | N/A | N/A | N/A | N/A |
| 127 | N/A | N/A | N/A | N/A | N/A | N/A | N/A | N/A | N/A | N/A | N/A | N/A | N/A | N/A | N/A |
| 128 | N/A | N/A | N/A | N/A | N/A | N/A | N/A | N/A | N/A | N/A | N/A | N/A | N/A | N/A | N/A |
| 129 | N/A | N/A | N/A | N/A | N/A | N/A | N/A | N/A | N/A | N/A | N/A | N/A | N/A | N/A | N/A |
| 130 | N/A | N/A | N/A | N/A | N/A | N/A | N/A | N/A | N/A | N/A | N/A | N/A | N/A | N/A | N/A |
| 131 | N/A | N/A | N/A | N/A | N/A | N/A | N/A | N/A | N/A | N/A | N/A | N/A | N/A | N/A | N/A |
| 132 | N/A | N/A | N/A | N/A | N/A | N/A | N/A | N/A | N/A | N/A | N/A | N/A | N/A | N/A | N/A |
| 133 | N/A | N/A | N/A | N/A | N/A | N/A | N/A | N/A | N/A | N/A | N/A | N/A | N/A | N/A | N/A |
| 134 | + | N/A | + | - | - | R | 0 | 12 m | N/A | N/A | N/A | N/A | N/A | N/A | N/A |
| 135 | + | N/A | + | - | - | R | 0 | 12 m | N/A | N/A | N/A | N/A | N/A | N/A | N/A |
| 136 | + | N/A | + | - | - | R | 0 | 12 m | N/A | N/A | N/A | N/A | N/A | N/A | N/A |
| 137 | + | N/A | + | - | - | R | 0 | 9 m | N/A | N/A | N/A | N/A | N/A | N/A | N/A |
| 138 | + | + | + | + | R, T | R | 1 | 17m | N/A | N/A | N/A | N/A | N/A | N/A | N/A |
| 139 | + | N/A | + | - | A, C, R | N | 0 | 24 m | N/A | N/A | N/A | N/A | N/A | N/A | N/A |
| 140 | + | + | + | - | - | CR | 0 | 5 m | N/A | N/A | N/A | N/A | N/A | N/A | N/A |
| 141 | + | + | + | - | - | CR | 0 | 5 m | N/A | N/A | N/A | N/A | N/A | N/A | N/A |
| 142 | + | + | + | - | T | CR | 0 | 12 m | N/A | N/A | N/A | N/A | N/A | N/A | N/A |
| 143 | + | + | - | - | - | CR | 0 | 3 m | N/A | N/A | N/A | N/A | N/A | N/A | N/A |
| 144 | + | + | - | - | A | CR | 1 | 6 m | N/A | N/A | N/A | N/A | N/A | N/A | N/A |
| 145 | - | N/A | - | - | - | N/A | 0 | N/A | N/A | N/A | N/A | N/A | N/A | N/A | N/A |
| 146 | + | + | + | - | - | R | 0 | 4 m | N/A | N/A | N/A | N/A | N/A | N/A | N/A |
| 147 | + | + | - | - | - | CR | 0 | 3 m | N/A | N/A | N/A | N/A | N/A | N/A | N/A |
| 148 | + | + | - | - | - | CR | 0 | 2 m | N/A | N/A | N/A | N/A | N/A | N/A | N/A |
| 149 | + | N/A | - | - | - | N/A | 0 | N/A | N/A | N/A | N/A | N/A | N/A | N/A | N/A |
| 150 | + | + | + | - | R | CR | 0 | 1 m | N/A | N/A | N/A | N/A | N/A | N/A | N/A |
| 151 | + | + | + | - | - | CR | 0 | 1 m | N/A | N/A | N/A | N/A | N/A | N/A | N/A |
| 152 | 30/38 | N/A | 20/38 | | - | CR 14/21, R 2/21,D 5/21 | 2/21 | ≥24 m | N/A | N/A | N/A | N/A | N/A | N/A | N/A |
| 153 | 44/76 | N/A | 26/76 | - | 14/76 (R 5, C 3, A 6, M 1) | CR 34/48, D 2/48 | 13/48 | ≥18 m | N/A | N/A | N/A | N/A | N/A | N/A | N/A |
| 154 | 16/16 | N/A | 7/16 | 5/16 | A 5/16 | R 13, D 1 | 2/16 | 38 (26-76) m | N/A | N/A | N/A | N/A | N/A | N/A | N/A |
| 155 | 17/19 | 17/19 | 9/19 | - | - | CR 13, D 1 | 6/19 | 1 m | N/A | N/A | N/A | N/A | N/A | N/A | N/A |
| 156 | 24/24 | 23/24 | 15/24 | - | - | R 3, D 0 | 0 | N/A | N/A | N/A | N/A | N/A | N/A | N/A | N/A |
| 157 | 7/9 | N/A | 4/9 | - | - | CR 2, R 5 | 0 | 16 m | N/A | N/A | N/A | N/A | N/A | N/A | N/A |
| 158 | 42/50 | N/A | 31/50 | 3/50 | 6/50 (R 3, A 2, Cs 1) | CR12, R 27, N 8, D 3 | 6/33 | 18 (2-60) | N/A | N/A | N/A | N/A | N/A | N/A | N/A |
| 159 | N/A | N/A | N/A | N/A | N/A | N/A | N/A | N/A | N/A | N/A | N/A | N/A | N/A | N/A | N/A |

Abbreviations: N/A: not applicable. IVIG: Intravenous immunoglobulin; PE: Plasma exchange. *d: ≥250mg/day. *e: A: Azathioprine. C: Cyclophosphamide. M: Mycophenolate mofetil. R: Rituximab. T: Tacrolimus. Cs: Cyclosporine. *f: R: Remission. CR: Complete remission. N: Not remission. D: death. r: relapse. *g: w: weeks. m: months.

**Supplementary Table 3. Combined therapy in cases with complete reports.**

|  | CR  n/N （%） | R  n/N （%） | CR+R  n/N （%） | r  n/N（%） | N/A |
| --- | --- | --- | --- | --- | --- |
| S+PE | 2/7 (28.57%) | 3/7 (42.86%) | 5/7 (71.42%) | 2/7 (28.57%) | 2 |
| S+I | 1/2 (50.00%) | 1/2 (50.00%) | 2/2 (100.00%) | 2/2 (100.00%) | 0 |
| S+IVIG+PE | 0/2 (0%) | 2/2 (100.00%) | 2/2 (100.00%) | 1/2 (50.00%) | 0 |
| S+IVIG+I | 3/9 (33.33%) | 5/9 (55.56%) | 8/9 (88.89%) | 1/9 (11.11%) | 1 |
| S+PE+I | 1/2 (50.00%) | 1/2 (50.00%) | 2/2 (100.00%) | 1/2 (50.00%) | 0 |
| S+IVIG+PE+I | 0/1 (0%) | 1/1 (100.00%) | 1/1 (100.00%) | 1/1 (100.00%) | 0 |

Abbreviations: CR: complete remission. R: remission. r: relapsed. N/A: not applicable. S: steroids. IVIG: intravenous immunoglobulin. PE: plasma exchange. I: immunosuppressant.

Data reported as n/N (%), where N is the total number of patients reported with treatment in each therapy and n is the number of patients in each outcome with specific therapy.
